# LAVA: An Open-Source Approach To Designing LAMP (Loop-Mediated Isothermal Amplification) DNA Signatures

**DOI:** 10.1186/1471-2105-12-240

**Published:** 2011-06-16

**Authors:** Clinton Torres, Elizabeth A Vitalis, Brian R Baker, Shea N Gardner, Marisa W Torres, John M Dzenitis

**Affiliations:** 1 Lawrence Livermore National Laboratory, Livermore, California, USA

## Abstract

**Background:**

We developed an extendable open-source Loop-mediated isothermal AMPlification (LAMP) signature design program called LAVA (LAMP Assay Versatile Analysis). LAVA was created in response to limitations of existing LAMP signature programs.

**Results:**

LAVA identifies combinations of six primer regions for basic LAMP signatures, or combinations of eight primer regions for LAMP signatures with loop primers, which can be used as LAMP signatures. The identified primers are conserved among target organism sequences. Primer combinations are optimized based on lengths, melting temperatures, and spacing among primer sites. We compare LAMP signature candidates for *Staphylococcus aureus *created both by LAVA and by PrimerExplorer. We also include signatures from a sample run targeting all strains of *Mycobacterium tuberculosis*.

**Conclusions:**

We have designed and demonstrated new software for identifying signature candidates appropriate for LAMP assays. The software is available for download at http://lava-dna.googlecode.com/.

## Background

Loop-mediated isothermal amplification (LAMP) is a DNA amplification technique with high specificity, efficiency, and speed, performed under isothermal conditions [1]. We are using LAMP to perform highly sensitive and specific detection of blood-borne pathogens with a new point-of-care instrument that is in development, targeting pathogens such as *Staphylococcus aureus, Pseudomonas aeruginosa*, and *Streptococcus pneumoniae*. This approach has the potential to operate at significantly lower cost than TaqMan PCR detection because it can be performed with less expensive materials and equipment [2,3].

The most common method for designing LAMP primers is to use PrimerExplorer V4 from Eiken Chemical Co. Ltd. http://primerexplorer.jp/e/index.html. While PrimerExplorer is very useful for LAMP signature design, as demonstrated by its widespread use, it has several limitations that reduced its usability for our high-throughput whole-genome analysis. First, PrimerExplorer doesn't support IUPAC characters other than "ATCG" in the input sequence, which are often used in MSA representations, because it was not built to handle MSA representations. Second, PrimerExplorer only runs in Windows operating systems, in a specific web browser. Third, PrimerExplorer cannot design signatures with loop primers, as discussed in Nagamine et al. [4] in a single execution, instead requiring two serial executions, which can prevent more optimal primer combinations from being identified. And fourth, PrimerExplorer is less suited for high throughput analysis since it is limited to a single execution process on a computer, accepts only up to 2,000 bp sequences, and outputs only HTML.

**Figure 1:**
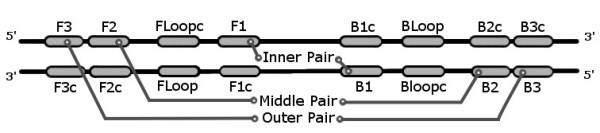
**Additional description of a LAMP signature**. Each named pair refers to a sequence location corresponding to the primer regions of like-numbered primers. These pairs represent the location and orientation of the primers with respect to the target template during each extension in which they participate.

LAVA is designed to be a flexible tool for custom signature design, so it can fulfill varying signature design needs in a high-throughput informatics environment. LAVA was implemented in Perl because Perl interpreters are available for every major operating system, the wide use of BioPerl [5] in bioinformatics, and BioPerl's support for several different sequence alignment formats. To simplify discussion of signature design, we refer to LAMP primers as pairs of nested primers: inner, loop, middle, and outer, as shown in Figure 1. All signature results from LAVA are read in the 5' to 3' direction, even if the opposite strand is used to design a portion of the sequence, and are consistent with the traditional nomenclature in Notomi et al. [1].

## Implementation

**Figure 2:**
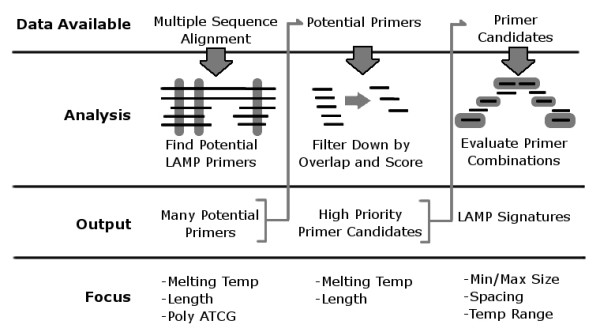
**Schematic diagram of LAVA**. MSAs are used as input. Potential primers are found based on the MSA content, and paired in all reasonable configurations as inner primers. The remaining primers are then selected based on the scoring of potential combinations.

This is a Multiple-Sequence Alignment (MSA) approach to LAMP signature design. LAVA's processing steps are outlined in Figure 2. Either a single sequence or a pre-computed MSA can be used as input. The individual sequences from the MSA are used for primer enumeration, and design parameters can be adjusted for each primer role. Once potential primer locations are found, they are usually down-selected based on overlap and relative score, which keeps the number of analyzed combinations down. After primers have been identified that are shared among all the individual sequences, combinations of primers for the roles of inner, loop, middle, and outer, are analyzed to select LAMP signature candidates.

### Find Potential LAMP Primers

MSAs are read as input with BioPerl's Bio::AlignIO module, which understands many different alignment formats. Long MSAs can sometimes be used as input, but have heavy computer resource requirements, and using long MSAs can result in fewer identified signature candidates.

LAMP signatures that cover individual non-MSA sequences 20 kbp in length can take up to 90 minutes on a desktop computer if few design constraints are specified. Identification of signatures for MSAs of this type is usually finished in minutes, because only regions of conservation are considered in this version of LAVA. MSAs can have both longer and shorter runtimes, depending on the content of the MSA. MSAs approaching 100% conservation will run similar to single sequences. As the level of conservation decreases, so will LAVA's runtime up to a point.

We suggest using the LAVA wrapper SLAVA (Serial-LAVA) for individual sequences and highly conserved MSAs over 10 kbp. SLAVA splits the MSA into sections, executes LAVA for each individual section, and combines the results into a single set of non-identical signatures. Running a series of smaller chunks through SLAVA is likely to result in more optimal signature combinations, because each sub-analysis can identify more primer candidate regions.

Primer enumeration is performed by modules which implement the OligoEnumerator interface. By default, this is done with Primer3 [6], executed through BioPerl's Bio::Tools::Run::Primer3 module. The primer search is separately run for each primer context of inner, loop, middle, and outer. Default primer design parameters, which can be individually customized, were chosen based on our primer design rules for other primer-based amplification techniques [7]. LAVA's default primer design parameters are listed in Table 1, along with other relevant parameter defaults discussed in this section. These defaults should only be considered as a starting point for signature identification, because a wide range of parameters yield successful LAMP assays.

**Table 1:** Default values of the most commonly adjusted LAVA parameters

Parameter	Default Target
Outer primer length	18-23 bp

Middle primer length	18-23 bp

Loop primer length	18-23 bp

Inner primer length	20-26 bp

Outer primer T_m_	59-61°C

Middle primer T_m_	59-61°C

Loop primer T_m_	58-62°C

Inner primer T_m_	62-66°C

Maximum signature length	320 bp

Minimum spacing from middle to inner primers	25 bp

Maximum consecutive repeated bases	5

The first sequence in the MSA is the basis for generating primers. The remaining MSA sequences are used to filter out primers that are not identically present in every target sequence. Sub-sequence with "N" or "-" characters is not considered a valid primer target. Primers that are shared among all MSA sequences are returned as potential signature components by the OligoEnumerator. During primer generation, a maximum poly-base restriction is enforced, limiting number of consecutive identical bases in a potential primer region.

Primer analysis and scoring is performed by PrimerAnalyzer modules. Penalties get applied to primers and combinations of primers in two separate places. First is as an individual primer, and second is as a combination of primers for a LAMP signature. The PrimerAnalyzer penalty for individual primers in this version of LAVA is simply the Primer3 penalty score, which reflects how closely the primer comes to the design parameters. For combinations of primers, the penalty also includes factors for for inter-primer spacing. The assessment of the primer by the PrimerAnalyzer is returned as PrimerInfo objects, which are used to provide primer sequence information for signature output. This may appear redundant because the primers already contain the original sequence information, but since different scores can exist for the same primer in different roles, and some analysis methods may impose context-sensitive sequence restrictions as part of score calculation, relying on PrimerInfo guarantees that the correct sequence is associated with the analysis result for each context.

### Filter Down By Overlap And Score

To help control the number of primer combinations that need to be scored, the set of candidate primer regions is down-selected based on overlap. Many potential primers often target the same general sequence region. Of the available primers, the best scoring primers are given priority during down-selection. The lower scoring overlapping primers are removed from consideration if they overlap the higher priority primers by a given percentage.

### Evaluate Primer Combinations

Finally, primers are combined into nested sets that can serve as LAMP signatures. The overall LAMP signature penalty is the weighted combination of inner, middle, and outer pair penalties, plus context-dependent spacing penalties. Inter-primer spacing penalty increases as distance increases. The default objective function includes slightly decreasing weights for the penalties of inner primers, middle primers, outer primers, and loop primers respectively.

If the minimum number of signatures is not identified for the target, the entire primer combination process is repeated with different primer overlap cutoff percentages. The amount of overlap permitted is set for each iteration, based on a "schedule" of primer overlap percentages. Since these repeat attempts are effectively multiple runs of LAVA, often with more individually considered primers, regions with difficult to identify signatures will take longer to process. Processing time can increase exponentially as the primer overlap restrictions become lighter.

## Results

We created LAMP signatures with both LAVA and PrimerExplorer for comparison. The locus we targeted is an 800 base-pair long sequence of *Staphylococcus aureus*, starting at base 2464089 of the RF122 genome [GenBank: NC_007622.1] at the 3' end of *gltA *through the 5' start of *gltB*. This locus is interesting to us because it is a place where a KPATH [7] run identified a TaqMan signature candidate that is both conserved among all targets, and is unique to the targets compared to all other known sequence, which makes this candidate region potentially valuable for *S. aureus *detection. Input for signature design was a sequence representation of all the genome sequences in Table 2.

**Table 2:** Strain sequences used for LAMP signature comparison between LAVA and PrimerExplorer

GenBank GI Number	GenBank Accession Number	Sequence Description
82749777	NC_007622.1	Staphylococcus aureus RF122, complete genome

148266447	NC_009487.1	Staphylococcus aureus subsp. aureus JH9, complete genome

150392480	NC_009632.1	Staphylococcus aureus subsp. aureus JH1, complete genome

57634611	NC_002758.2	Staphylococcus aureus subsp. aureus Mu50, complete genome

29165615	NC_002745.2	Staphylococcus aureus subsp. aureus N315, complete genome

21281729	NC_003923.1	Staphylococcus aureus subsp. aureus MW2, complete genome

88193823	NC_007795.1	Staphylococcus aureus subsp. aureus NCTC 8325, complete genome

57650036	NC_002951.2	Staphylococcus aureus subsp. aureus COL, complete genome

49482253	NC_002952.2	Staphylococcus aureus subsp. aureus MRSA252, complete genome

49484912	NC_002953.3	Staphylococcus aureus subsp. aureus MSSA476, complete genome

87159884	NC_007793.1	Staphylococcus aureus subsp. aureus USA300, complete genome

161508266	NC_010079.1	Staphylococcus aureus subsp. aureus USA300_TCH1516, complete genome

151220212	NC_009641.1	Staphylococcus aureus subsp. aureus str. Newman, complete genome

156978331	NC_009782.1	Staphylococcus aureus subsp. aureus Mu3, complete genome

Eiken's PrimerExplorer was run with default parameters. LAVA was run with parameters designed to be similar to the PrimerExplorer defaults, but with an adjusted melting temperature target range to compensate for the difference in calculated temperatures between Primer3 and PrimerExplorer. These parameters specifically allow a wider acceptable TM range from LAVA's defaults, and a longer poly-base limit. The best scoring signature results from both programs are provided in Table 3.

**Table 3:** LAMP signature candidate regions for *S. aureus*, as generated by both LAVA and PrimerExplorer. T_m _calculated with BioPerl using calculations from SantaLucia(8) with 50 mg/L salt concentration and 50 ng/L oligo concentration

Program	Primer	Sequence	T_m _(C)	5' Location	Length
LAVA	F1	GGAATAGTTTGTAAGACACCTGCCA	55.82	149	25

	F2	ACCAACACCAAAAATCGGT	50.22	103	19

	F3	GCTACAATTGCAGGCGTTT	51.66	83	19

	B1	CAAAAACAAAGCGAACTGCCAAT	54.18	209	23

	B2	TGGCATTATTACTTGCCATCA	50.38	260	21

	B3	TTGATGTCGAAAACACTGGAA	50.63	300	21

PrimerExplorer	F1	TGTTGGAATAGTTTGTAAGACACCT	53.22	145	25

	F2	TTACCAACACCAAAAATCGG	48.91	101	21

	F3	GCTACAATTGCAGGCGTT	50.80	83	18

	B1	CAAAAACAAAGCGAACTGCCAATA	53.99	209	24

	B2	GCATTATTACTTGCCATCATTG	48.63	258	22

	B3	TGTCGAAAACACTGGAACAT	50.11	296	20

**Figure 3:**
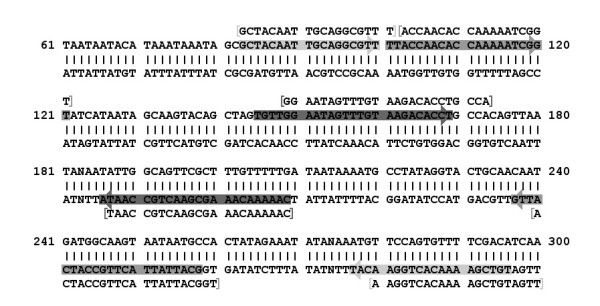
**Signature layout of PrimerExplorer and LAVA results**. This shows results for the specified S. aureus sequence locus, where 'N' characters represent bases not perfectly conserved across all targets. PrimerExplorer results are highlighted, and LAVA results are repeated above or below the double-stranded sequence as appropriate.

The LAVA selection for a lamp signature is nearly identical to the PrimerExplorer selection, as seen in Figure 3. We believe the variations between result sets are the result of subtle differences in calculation methods for primer metrics, and would not represent a significant difference in signature behavior. It is likely that LAVA penalizes T_m _differences slightly more than PrimerExplorer. The result is that LAVA's top signature selection has a slightly smaller range of melting temperatures.

As an additional example case, we've also created LAMP signature candidates for Mycobacterium tuberculosis, as listed in Table 4, using default LAVA parameters. We have not screened these in the laboratory, but they represent conserved and unique signatures that we believe would make sensitive and specific detection assays, similar to some of the assays in Iwamoto et al. [9]. A pre-computed MSA of all 25 completed Mycobacterium tuberculosis genomes available was used as input, including known drug-resistant genomes, and non-unique regions were masked out so only unique sequence is used for signature design.

**Table 4:** LAMP signature candidates for *Mycobacterium tuberculosis*, with gene targets based on the reference H37Rv genome [GenBank: NC_000962.2]. The hyphen in FIP and BIP sequences represents where the two segments should be linked together

Signature	Size	Gene	Part	Sequence	Length
mTub228	275 bp	Rv0987	F3	CGCTCTCAGTTTGATTGCCT	20 bp

			B3	CATTGCGAACATTGATGACA	20 bp

			FLoop	CTCGGGTTACACCCAAACAG	20 bp

			BLoop	CTTGATTGGAATTTGGCTCG	20 bp

			FIP	GGATTCCGTCATTATCAGCCAAA-GACCGTTTTTCGCCATATTG	43 bp

			BIP	CTGATTGGTACGGGCTTGGG-TGATGGTTTGAGTCACCAGG	40 bp

mTub229	195 bp	Rv0988	F3	AATGGCACCGCTTTGATG	18 bp

			B3	AATTTCGATTTCCCATGCAA	20 bp

			FLoop	CTGGAAAATCGGGTCACACT	20 bp

			BLoop	ATTTCCGCATCAAGACGACT	20 bp

			FIP	CATCCCCGGCAACAAAGGTA-TTTTCAGATTCGACAGGCG	39 bp

			BIP	GTGATCCCTCTCGAGTCGTCC-CACTCTGGTCACTGGTCCAA	41 bp

mTub230	252 bp	Rv1290c	F3	GACACCACGATCAGCACG	18 bp

			B3	CGTAGTTCCGCCTAGTGCTC	20 bp

			FLoop	GTCGATGATTCCCGTGAAAT	20 bp

			BLoop	ACGGTTGAGCATGCTGGT	18 bp

			FIP	AGTCTGGGTGCTGCCGACAT-GCCCTGAAACATCAGCTTGT	40 bp

			BIP	CGCCCTGTAAGTAATCCAGTATGG-GGATGGGTGCATGCTTATTC	44 bp

mTub231	235 bp	tyrS (approx)	F3	GATTGGGAGGTGATGAGACC	20 bp

			B3	CTAGTCGTGGGATACCAGCG	20 bp

			FLoop	CGTACTCGATCCCGTTGTTC	20 bp

			BLoop	TGCGTTGTAGTCGATTTCCA	20 bp

			FIP	TCACACGCACAGCTGTTTAGTGA-TAGTGCGTTGACCTCACTCG	43 bp

			BIP	CATCATCCTTTCATGTGACAGGC-CCGCCAAAGATAAGTCAGGA	43 bp

mTub232	230 bp	Rv2735c and recX	F3	CGGTCTATGTTCTCGGGCT	19 bp

			B3	GTGGGTACGGCCAGACCT	18 bp

			FLoop	GCCTTCAACAGGGCTAGTCA	20 bp

			BLoop	CATCGAATCCCTTTAGACGC	20 bp

			FIP	AACAAACTGGAGATACTTGCCGG-GTCGAGGTAAATTCGTTCACG	44 bp

			BIP	CGCGTCCAATATGACCATTCTCTA-GTGGTTATCGCCGAGCTG	42 bp

## Discussion

When developing LAVA, getting usable LAMP signatures was our priority, so we have not spent time on optimizations, or on many of the features we desire. Operationally, LAVA has fulfilled our needs, but there are several components that are worthy of further attention. This discussion explores aspects of LAVA's design and operation that we believe will improve or augment LAVA's performance the most.

LAVA currently excludes dimerization checks during primer selection, which would contribute to the relative scores of signature candidates. This would have been accomplished using the UNAFold [10] libraries to analyze inter-oligo interactions, and primer self-annealing. This is currently omitted because in some instances, the increase in required processing time to analyze primer combinations was unacceptable. Predicting inter-oligo hybridization is still a serious concern with regards to LAMP signature design, so we separately perform dimerization analysis on the finished signature candidates, and avoid using candidates with high dimerization potential. We will include dimerization checks as part of the native signature design when possible.

There are two primary options for controlling the number of primer combinations that need to be analyzed. The first is limiting the number of primer candidates that Primer3 can identify in each execution. This can be accomplished by narrowing the acceptable range of primer design parameters such as length and melting temperature. Primer3 output can also be limited by explicitly setting a maximum number of primers for Primer3 to generate. The second option is to set stricter primer overlap limits. Stringent overlap limits may make it possible to perform the comprehensive dimerization checks discussed above within reasonable processing times.

We have observed through computer predictions including [10], that an optional linker sequence, used to connect the two components of the FIP and BIP (F1c and F2, and B1c and B2 respectively), has the potential to disrupt the LAMP reaction. In general, calculations based on a "TTTT" linker sequence predict a slight increase in sensitivity in many of the likely hybridization configurations, because of a slightly longer and more stable base pairing at the 5' end of the hybridization. However, in one instance, this linker greatly increased predicted primer self-hybridization because of an unfortunate co-incidence of self-similar sequence. The chance of this being a problem increases if the number of consecutive identical bases in the designed primers cannot be limited. Improper choice of linker sequence can also increase predicted primer dimerization. A more context-aware linker design should yield better results than always using a single linker sequence. One day, we hope LAVA will suggest the most appropriate linker sequence, or omission of the linker, for each designed signature, to help support desired assay behavior. One potential approach is to design linkers that are the least complimentary to the loop regions adjacent to the F1 region in the LAMP dumbbell structures as possible. Another potential approach is to design linker-free primers like Poon et al [11], which is currently the default behavior of LAVA.

When designing LAMP signatures, we found there is a general pattern we fell into of relaxing design parameters to identify signatures for difficult to target sequences. On average, the order which we adjusted parameters were to increase melting temperature range, decrease primer minimum length, and finally to increase signature max length.

The LAVA wrapper SLAVA was implemented as a serial execution of individual MSA segments. This is provided to enable signature design for long sequences such as whole bacterial genomes, and also to demonstrate how a parallel implementation would be structured. During the further development of LAVA, we hope to transition into a high-performance grid computing environment with a parallel LAVA implementation.

We are separately developing a OligoEnumerator for creating primers with degeneracy, which are primers with base variations designed to accommodate strain variation. So far, we have used a "masked" MSA representation of the target to design signatures. However, since Primer3 does not accept sequence containing the standardized IUPAC character codes, the MSA representation has so far been limited to perfect consensus sequence. The fundamental difference in approach for designing primers with degeneracy, is that primers are enumerated based on all the sequences of the MSA, instead of based on only the first sequence. Our proof of concept requires different internal representations of sub-sequence MSAs, but is built using the existing OligoEnumerator and PrimerAnalyzer interfaces.

## Conclusions

We have designed and demonstrated new software for identifying signature candidates appropriate for LAMP assays. LAVA is available as open source, downloadable from the project home page. The focus of LAVA is to improve on other currently available software by accommodating high-throughput signature design, while providing a framework to develop more sophisticated algorithmic and analytical tools. We have used LAVA to design LAMP signatures for several organisms, which are currently undergoing bench screening and optimization for use in a point-of-care detection instrument.

## Availability and Requirements

Project name: LAVA-DNA

Project home page: http://lava-dna.googlecode.com/

Operating system: Unix/Linux

Programming language: Perl

Other requirements:

BioPerl 1.5.2 or higher [http://www.bioperl.org/]

Primer3 1.0 or higher [http://primer3.sourceforge.net/]

License: BSD

## Authors' contributions

CT wrote the LAVA software. EAV oversaw the pathogen informatics. BRB and JMD helped define the optimal assay constraints. SNG and MWL contributed to the LAVA architecture and design. All authors read and approved the final manuscript.
